# Phase II multicenter clinical trial of hypoallergenic 1BS-18 Hokushin bread oral immunotherapy for wheat-dependent exercise-induced anaphylaxis

**DOI:** 10.5415/apallergy.0000000000000180

**Published:** 2025-02-05

**Authors:** Kunie Kohno, Yuko Chinuki, Akiko Sugiyama, Reiko Kishikawa, Mayumi Okamoto, Michihiro Hide, Yoshiko Oda, Atsushi Fukunaga, Ritsuro Suzuki, Eishin Morita

**Affiliations:** 1 Department of Dermatology, Shimane University Faculty of Medicine, Shimane, Japan; 2 Clinical Research Center, Shimane University Hospital, Shimane, Japan; 3 Department of Allergology, National Hospital Organization Fukuoka National Hospital, Fukuoka, Japan; 4 Department of Dermatology, Institute of Biomedical and Health Sciences, Hiroshima University, Hiroshima, Japan; 5 Division of Dermatology, Department of Internal Related, Kobe University Graduate School of Medicine, Hyogo, Japan; 6 Division of Medicine for Function and Morphology of Sensory Organs, Department of Dermatology, Faculty of Medicine, Osaka Medical and Pharmaceutical University, Osaka, Japan

**Keywords:** ω5-gliadin, Basophil activation test, desensitization, hypoallergenic wheat, wheat-dependent exercise-induced anaphylaxis

## Abstract

**Background:**

Therapies for desensitizing wheat-dependent exercise-induced anaphylaxis (WDEIA), a severe allergic response to wheat ingestion and exercise, remain unestablished. This study aimed to investigate whether continuous ingestion of hypoallergenic 1BS-18 Hokushin bread, which lacks the *Gli-B1* locus encoding the ω5-gliadin allergen, could achieve desensitization in adult patients with WDEIA sensitized to ω5-gliadin.

**Methods:**

Sixteen adult patients diagnosed with WDEIA participated in this study. Each patient was administered a safety dose of bread made from 1BS-18 Hokushin wheat, a hypoallergenic wheat that defects the *Gli-B1* locus responsible for encoding the ω5-gliadin allergen, over a 12-week period. The safe dose for each individual was determined through a stepwise increase in bread intake and monitored to prevent allergic reactions. Desensitization efficacy was evaluated by measuring basophil activation rates and serum allergen-specific IgE levels specific to wheat proteins using the basophil activation test and ImmunoCAP serum testing.

**Results:**

Fourteen of the 16 patients (87.5%) successfully completed the 12-week regimen of 1BS-18 Hokushin bread, with 2 patients (12.5%) discontinuing due to allergic reactions associated with the bread. Evaluation of basophil activation rates and serum allergen-specific IgE levels indicated no significant desensitization effects in any patient.

**Conclusions:**

Approximately 80% of patients with WDEIA were able to safely consume 1BS-18 Hokushin bread at least up to 60 g per day for 12 weeks without severe adverse reactions. However, this regimen did not achieve desensitization, suggesting that further studies may be necessary to explore alternative dosing, duration, or combinations with adjunct therapies for effective desensitization in patients with WDEIA.

## 1. Introduction

IgE-mediated wheat allergy causes allergic symptoms, such as urticaria, angioedema, abdominal pain, diarrhea, and anaphylactic shock, when wheat products are ingested. Wheat-dependent exercise-induced anaphylaxis (WDEIA) is a form of IgE-mediated wheat allergy that predominantly develops after school age or in adults and typically shows allergic symptoms in combination with wheat ingestion and cofactors, such as exercise or nonsteroidal anti-inflammatory drugs [1]. IgE-mediated wheat allergy in infancy mainly develops in association with atopic dermatitis and mostly acquires resistance by the age of 12 years [2], whereas WDEIA develops in adult individuals without atopic dermatitis and continues for a long period [3]. An epidemiological survey in Shimane prefecture has shown that the prevalence of WDEIA is 0.21% in adults [4].

Since therapies leading to the remission of WDEIA have not yet been established, the elimination of wheat products from the diet or avoidance of exercise after ingesting wheat products has been advised for patients with WDEIA. However, wheat is a widely consumed food, and strict restriction of wheat products in patients’ diets elicits a substantial decline in their quality of life. Our previous analytical study of the causative allergens of WDEIA revealed that ω5-gliadin is a major allergen among gluten proteins [5]. Based on the findings of this study, the *Chinese Spring* Chromosome mutant line 1BS-18, a natural wheat line with chromosomal defects lacking the *Gli-B1* locus, including the ω5-gliadin encoding region, was selected to develop wheat products that could be safely consumed by patients with WDEIA [6]. Low allergenicity of the 1BS-18 wheat was confirmed using a guinea pig challenge model [6]. Challenge tests with 100 g of bread made from 1BS-18 combined with exercise in 5 patients with WDEIA showed that 2 of the 5 patients were asymptomatic, and the other 3 patients developed urticaria, one of which required systemic antihistamines (unpublished). Because the 1BS-18 *Chinese spring* line is an experimental line and is unsuitable for practical use because of its low yield and gluten-forming ability, it has been repeatedly backcrossed with the edible wheat line Hokushin, resulting in the edible wheat line 1BS-18 Hokushin, which lacks the *Gli-B1* locus of 1B chromosome of wheat. The immunoreactive ω5-gliadin content of 1BS-18 Hokushin was 1.21 mg/g gluten, which was much lower than that of euploid Hokushin (5.17 mg/g gluten) [7]. Low allergenicity of 1BS-18 Hokushin wheat was confirmed using a wheat allergy rat model [8]. In addition, early consecutive ingestion of 1BS-18 Hokushin wheat prevented subcutaneous immunization against ω5-gliadin protein in the rat wheat-anaphylaxis model, suggesting that 1BS-18 Hokushin wheat induces oral tolerance to wheat allergens [9].

The objective of this study was to examine whether desensitization can be achieved by the continuous ingestion of 1BS-18 Hokushin bread in adult patients with WDEIA sensitized to ω5-gliadin who are under restriction of consumption of wheat products. Since 1BS-18 Hokushin bread is not completely free from the allergenic activity of WDEIA owing to the cross-reaction of the ω5-gliadin-specific IgE to other gluten proteins possessing ω5-gliadin-epitopes or the possible existence of the low amount of ω5-gliadin protein derived from the 1D chromosome [7], the amount of 1BS-18 Hokushin bread to be safely consumed was determined by a gradual incremental method from a small amount in each individual.

## 2. Subjects and methods

### 2.1. Subjects

A phase II multicenter clinical trial was conducted at 4 sites across Japan: Shimane University Hospital, Fukuoka National Hospital, Hiroshima University Hospital, and Kobe University Hospital. Sixteen patients with WDEIA who met the eligibility criteria were included in this study (Table 1). This study complied with the ethical principles of the Declaration of Helsinki. Written informed consent was obtained from each patient at the time of enrollment. The study was approved by the ethics committee of Shimane University Faculty of Medicine (approval no. 2537), together with the corresponding committees of the other three institutions, and registered in a public registry (UMIN000024951).

**Table 1.** Eligibility criteria

Inclusion criteria
(1) Subjects who were 20 years of age or older. (2) Subjects who met the diagnostic criteria by the study group of Health and Labour Sciences Research Grant (Supplementary Table 1, http://links.lww.com/PA9/A56) and have a history of allergic symptoms due to consumption of wheat products within 1 year before registration, or restricting consumption of wheat products due to high serum level of ω5-gliadin-specific IgE (>0.35 UA/mL) tested within 4 weeks before registration. (3) Positive serum ω5-gliadin-specific IgE test (>0.1 UA/mL). (4) Subjects can visit every 1–4 weeks. (5) Written personal consent has been obtained for this study.
Exclusion criteria
(1) Patients taking antihistamines (allergy symptoms may be suppressed and not accurately assessed) and nonsteroidal anti-inflammatory drugs (which may exacerbate allergic symptoms). (2) Patients with immediate allergy to milk products (because 1BS-18 Hokushin bread used in clinical research contains small amounts of milk products). (3) Patients who received drugs with immunosuppressive agents, including high-dose steroids, within 3 months before enrollment. (4) Patients with decompensated cirrhosis. (5) Patients with serious renal disease (latest serum Cr of 2.0 mg/dL or higher within 4 weeks before enrollment). (6) Pregnant and lactating women or women who may be pregnant. (7) Other patients deemed unsuitable by their physician for participation in this study.

### 2.2. Preparation of 1BS-18 Hokushin bread

Hypoallergenic bread was prepared using the 1BS-18 Hokushin wheat line, which was developed through repeated backcrossing between Hokushin (a variety widely cultivated for wheat products in Japan) and the natural defects wheat line 1BS-18 *Chinese Spring* (established as a hypoallergenic wheat line lacking *Gli-B1* by Kohno et al.) [6, 8]. The fundamental components were as follows: The recipe calls for 250 g of 1BS-18 Hokushin wheat flour, 15 g of unsalted butter, 17 g of white sugar, 6 g of skim milk, 5 g of salt, 2.8 g of dry yeast, and 180 g of water. The bread was prepared using fully automatic bread makers (SD-BH101; National, Tokyo, Japan; and SD-MT-2; Panasonic, Tokyo, Japan).

### 2.3. Ingestion schedule of 1BS-18 Hokushin bread for desensitization

**Figure 1.**
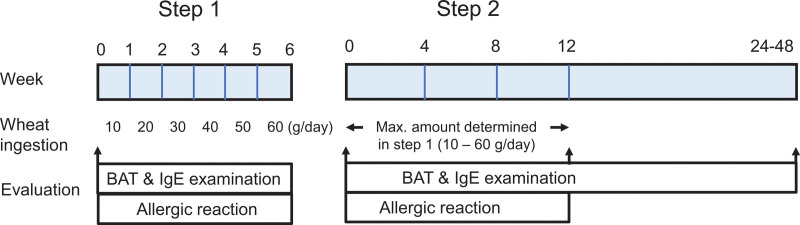
Schedule for the ingestion of 1BS-18 Hokushin bread for desensitization. A total of 16 subjects followed the prescribed ingestion schedule for the bread. In the initial phase, the quantity of bread was incrementally augmented from 10 g/d to 60 g/d, with the objective of ascertaining the optimal dosage of 1BS-18 bread. In the second step of the procedure, the subjects were instructed to ingest the quantity of 1BS-18 Hokushin bread that had been determined to be safe in the preceding step on a continuous basis. BAT, basophil activation test.

Before starting desensitization, a safe consumed dose of 1BS-18 Hokushin bread, which the patient could ingest without experiencing allergic reactions in their daily life allowing exercise, was determined for each patient (Fig. 1, step 1). The amount of bread ingested was started at 10 g/d and continued for 1 week if no allergic symptoms were observed. The amount of bread was increased from 10 g/d for a week to 60 g/d for a week with an increase of 10 g/d each. Ingestion was stopped when allergic symptoms were observed, and a maximum dose of up to 60 g/d without allergic symptoms was determined to be safe for the patient. In the desensitization study, the safe consumed dose of 1BS-18 Hokushin bread assessed in step 1 was ingested every breakfast for 12 weeks (Fig. 1, step 2). During steps 1 and 2, patients were prohibited from consuming wheat products, except for 1BS-18 Hokushin bread. After completing step 2, serum allergen-specific IgE and basophil activation in response to wheat allergens were examined. The serum allergen-specific IgE levels and basophil activation were monitored for up to 48 months. Antihistamines, high-dose corticosteroids, immunomodulatory therapies, and other therapies that affected the evaluation in this study were not administered during the study, except for emergency use.

### 2.4. Evaluation of allergic symptoms, serum allergen-specific IgE test, and basophil activation test

Allergic symptoms were evaluated according to the Anaphylaxis Guidelines with slight modifications (Supplementary Table 1, http://links.lww.com/PA9/A56) [10]. Allergen-specific IgE levels in wheat, gluten, and ω5-gliadin were determined using ImmunoCAP (Thermo Fischer Diagnostics, Tokyo, Japan). An allergen-induced CD203c expression-based basophil activation test was performed as previously described [10]. The wheat allergens to activate basophils were prepared from commercially available wheat flour as follows: ethanol extraction fraction of wheat protein (EtOH), which contains mainly gliadins (final concentrations 1 and 10 μg/mL), alkali extraction fraction of wheat protein (Alkali), which contains mainly glutenins (final concentrations 1 and 10 μg/mL), and purified ω5-gliadin (final concentrations 0.1 and 1 μg/mL). After activation with these agents, CD203c expression on the basophil surface was monitored using fluorescence-activated cell sorting. The activation rate was assessed in comparison to anti-IgE activation, and the final value was calculated as a percentage of the expression induced by anti-IgE activation.

### 2.5. Outcomes

The primary endpoint of this study was the desensitization of subjects to wheat allergens in their peripheral blood basophil activation test after a 12-week continuous intake of 1BS-18 Hokushin bread. A previous report showed that patients with tolerance to wheat displayed basophil activation rates lower than 10%, whereas patients with active wheat allergy displayed activation rates greater than 10% [11], desensitization was defined as a decrease in the basophil activation rate against wheat allergens to 10% or less in each enrolled patient. The secondary endpoint was to evaluate safety in the continuous ingestion of 1BS-18 Hokushin bread for 12 weeks: a percentage of all eligible subjects who had zero score for subjective and objective symptoms for 12 weeks after the commencement of ingesting a safe consumed dose of 1BS-18 Hokushin bread according to “Scoring system for evaluating allergic symptoms” in which maximum score is 16 (Supplementary Table 2, http://links.lww.com/PA9/A56).

### 2.6. Statistical analyses

Since the efficacy rate of tolerance induction by oral immunotherapy with wheat in pediatric cases was reported to be approximately 37% to 60% [12–14], the expected efficacy rate was assumed to be 30% to 60%. The lower limit for clinically recommended treatment for patients with WDEIA is approximately 30%, and the threshold success rate is set at 30%. To test these hypotheses with a one-sided α error of 5% and power of 80%, the number of eligible patients required for this study was calculated as 16 using a binominal distribution.

Changes in the basophil activation rate and serum allergen-specific IgE values were tested for significance using the paired Dunnett method, considering the nonnormal distribution. Statistical significance was set at *P* < 0.05. Statistical analyses were performed using the SPSS Statistics version 25 (IBM, Armonk, NY, USA).

## 3. Results

Sixteen patients with WDEIA were enrolled in this study. The patients’ baseline characteristics are presented in Table 2. The mean patient age was 51.4 years (range: 31–77 years). All 16 patients had positive ω5-gliadin-specific IgE (>0.10 UA/mL) and showed a positive basophil activation rate (>10%) with ω5-gliadin (final concentration 1 μg/mL). In step 1, the safe consumed dose of 1BS-18 Hokushin bread was determined to be 60 g in 14 patients (87.5%) and 40 g in 2 patients, SHIM010 and FUKU002 (12.5%) (Table 3).

**Table 2.** Baseline characteristics of the patients

Patients	Age	Gender	Allergen-specific IgE (UA/mL)			Basophil activation rate (% activation)					
			Wheat	Gluten	ω5-gliadin	EtOH 1	EtOH 10	Alkali 1	Alkali 10	ω5-gliadin 0.1	ω5-gliadin 1
SHIM010	61	Female	1.35	6.95	20.2	102.3	92.4	16.5	90.6	102.6	92.1
SHIM011	61	Male	0.48	5.22	23.1	56.2	51.4	71.4	49.2	78.1	46.0
SHIM012	44	Female	0.17	1.15	5.39	64.9	75.0	43.4	75.8	84.7	57.5
SHIM013	61	Male	0.14	0.71	3.93	79.5	87.5	89.4	96.7	13.3	98.6
SHIM014	51	Male	0.1	1.41	7.82	93.4	93.8	96.4	94.9	104.1	99.1
SHIM015	25	Female	<0.1	0.17	5.02	34.5	101.0	2.8	88.8	11.0	87.8
SHIM016	77	Male	<0.1	<0.1	0.14	29.2	35.7	7.6	42.3	22.6	35.5
SHIM017	49	Female	<0.1	0.61	4.61	78.0	94.9	91.7	96.6	94.7	81.8
HIRO007	42	Female	<0.1	0.39	2.16	86.6	77.4	71.6	87.8	29.9	89.6
HIRO009	31	Male	0.21	0.42	0.53	81.7	81.2	70.3	82.1	21.9	91.4
HIRO010	67	Male	<0.1	0.32	1.86	96.9	84.5	85.5	97.3	77.5	94.4
FUKU001	56	Male	0.11	0.30	6.62	9.6	10.8	0.0	37.6	0.0	35.1
FUKU002	45	Female	0.11	0.97	4.55	0.6	9.1	3.7	12.2	6.8	13.2
FUKU004	56	Male	0.14	0.62	11.8	76.3	86.7	61.2	87.7	73.3	94.7
FUKU005	48	Male	<0.1	<0.1	0.71	16.6	12.6	15.3	25.5	4.7	27.5
KOBE004	49	Male	0.13	0.22	1.32	45.4	51.8	28.5	67.8	47.8	62.5

ω5-gliadin 0.1, purified ω5-gliadin (final concentration 0.1 μg/mL); ω5-gliadin 1, purified ω5-gliadin (final concentration 1 μg/mL); Alkali 1, alkali extraction fraction of wheat protein (final concentration 1 μg/mL); Alkali 10, alkali extraction fraction of wheat protein (final concentration 10 μg/mL); EtOH 1, ethanol extraction fraction of wheat protein (final concentration 1 μg/mL); EtOH 10, ethanol extraction fraction of wheat protein (final concentration 10 μg/mL).

**Table 3.** Individual primary endpoint (patients who achieved basophil activation rate below 10% after step 2) and secondary endpoint (score of allergic reaction associated with wheat ingestion during step 2)

Patients	Step 1*	Step 2†	Allergy score‡	Basophil activation rate§		
				EtOH	Alkali	ω5-gliadin
SHIM010	40 g	Discontinued	3	-	-	-
SHIM011	60 g	Completed	0	-	-	-
SHIM012	60 g	Completed	0	+	+	-
SHIM013	60 g	Completed	0	-	-	-
SHIM014	60 g	Completed	0	-	-	-
SHIM015	60 g	Completed	0	-	-	-
SHIM016	60 g	Completed	0	-	-	-
SHIM017	60 g	Completed	0	-	-	-
HIRO007	60 g	Completed	0	-	-	-
HIRO009	60 g	Discontinued	2	-	-	-
HIRO010	60 g	Completed	0	-	-	-
FUKU001	60 g	Completed	0	-	-	-
FUKU002	40 g	Completed	0	-	-	-
FUKU004	60 g	Completed	0	-	-	-
FUKU005	60 g	Completed	0	-	-	-
KOBE004	60 g	Completed	0	-	-	-

* safe consumed dose of 1BS-18 Hokushin bread.

† Outcome of 12-week continuous ingestion of safe ingestion dose.

‡ Score of allergic reaction associated with 1BS-18 Hokushin bread ingestion during step 2.

§ Below 10% with each preparation was presented as +.

ω5-gliadin, purified ω5-gliadin; Alkali, alkali extraction fraction of wheat protein; EtOH, ethanol extraction fraction of wheat protein.

In step 2, 14 of the 16 patients (87.5%) completed the 12-week continuous ingestion of 1BS-18 Hokushin bread (FUKU002 with 40 g, the remaining 13 patients with 60 g), whereas 2 (SHIM010 with safe consumed doses of 40 g, HIRO009 with safe consumed doses of 60 g) of the 16 patients (12.5%) discontinued because of an allergic reaction associated with 1BS-18 Hokushin bread (Table 3). The allergic reaction scores were 3 (generalized urticaria with fatigue) for SHIM010 and 2 (generalized urticaria) for HIRO009. The allergic symptoms in both patients resolved with the intravenous administration of antihistamines.

**Figure 2.**
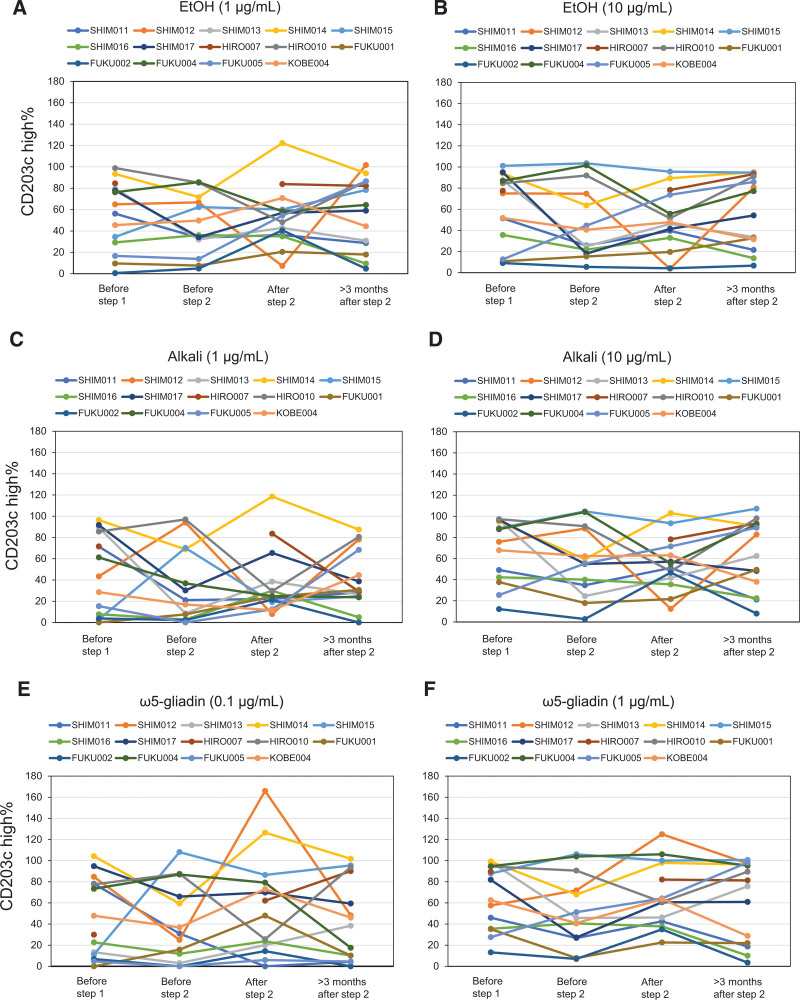
Basophil activation rates to each wheat preparation. Basophil activation rates were examined to observe the desensitization status of each wheat component in a total of 14 subjects throughout the trial period. (A) 1 μg/mL EtOH (ie, ethanol extraction fraction of wheat protein); (B) 10 μg/mL EtOH; (C) 1 μg/mL alkali (ie, alkali extraction fraction of wheat protein); (D) 10 μg/mL alkali; (E) 0.1 μg/mL ω5-gliadin; (F) 1 μg/mL ω5-gliadin. ω5-gliadin, purified ω5-gliadin; Alkali, alkali extraction fraction of wheat protein; EtOH, ethanol extraction fraction of wheat protein.

The primary endpoint was evaluated in 14 patients who completed step 2. One patient (SHIM012) (7.1%) achieved a basophil activation rate below 10% with EtOH and Alkali, whereas that with ω5-gliadin did not reach below 10% (Table 3). The other 13 patients did not achieve a basophil activation rate below 10% with all allergen preparations, indicating that a 12-week continuous intake of 1BS-18 Hokushin bread had little or no effect on basophil sensitivity to wheat allergens. The basophil activation rates from baseline to over 12 weeks of follow-up after continuous ingestion of 1BS-18 Hokushin bread in the 14 patients showed considerable variability among patients for each wheat preparation tested (EtOH 1 μg/mL, EtOH 10 g/mL, Alkali 1 μg/mL, Alkali 10 μg/mL, ω5-gliadin 0.1 μg/mL, and ω5-gliadin 1 μg/mL) (Fig. 2). Basophil activation rates with wheat preparations did not significantly change from the start to after 12 weeks of continuous intake of 1BS-18 Hokushin bread (Supplementary Table 3, http://links.lww.com/PA9/A56).

The secondary endpoint, evaluated as a safety after 12 weeks of continuous ingestion of 1BS-18 Hokushin bread in 16 patients, was achieved in 14 patients (87.5%). The allergen-specific IgE values for ω5-gliadin and gluten did not significantly change from baseline to over 12 weeks of follow-up after continuous ingestion of 1BS-18 Hokushin bread (Supplementary Table 4, http://links.lww.com/PA9/A56).

## 4. Discussion

We report the results of a phase II clinical trial of 12 weeks-continuous intake of the hypoallergenic 1BS-18 Hokushin bread. This study demonstrated the safety of continuous intake of 1BS-18 Hokushin bread in 87.5% of adult patients with WDEIA in daily life, including exercise; however, this treatment raised no signs of desensitization when evaluating the basophil activation rates and serum allergen-specific IgE values against wheat allergens. These results suggest that a continuous intake of hypoallergenic wheat at a safe dose for 12 weeks is not sufficient to achieve desensitization in adult patients with WDEIA; thus, higher maintenance doses and a longer immunotherapy course are required.

In the present study, the basophil activation rate was used to evaluate desensitization because previous reports showed that this parameter reflects the sensitization condition, such as degree and specificity, of patients with IgE-mediated wheat allergy [11, 15]. Patients with tolerance to wheat displayed basophil activation rates lower than 10%, whereas patients with active wheat allergy displayed activation rates greater than 10% [11]. The test clearly distinguished sensitization to allergens, ω5-gliadin, and hydrolyzed wheat proteins in patients with WDEIA [15]. In our previous study using the anti-IgE monoclonal antibody, omalizumab, most of the patients with WDEIA consumed wheat products without allergic reactions after their basophil activation rate with wheat allergens decreased to below 10% [10]. Furthermore, the basophil activation test is a safe and reliable ex vivo test to show sensitization conditions, because it can detect allergen-triggered cross-linking of IgE bound on the basophils in a specific manner.

In this study, both wheat gluten- and ω5-gliadin-specific IgE levels were monitored simultaneously as possible desensitization markers; however, no significant reduction was observed after 12 weeks of continuous bread ingestion in these IgE levels. Several clinical trials on oral immunotherapy using wheat products in children with wheat allergies have been conducted. However, the role of IgEs as biomarkers for desensitization remains controversial. Sugiura et al. [14] reported a significant decrease in the serum wheat- and ω5-gliadin-specific IgE levels in the oral immunotherapy group (41.7% tolerance) compared with the complete avoidance group (11.1% tolerance), indicating that wheat- and ω5-gliadin-specific IgE levels are biomarkers for achieving tolerance. In contrast, Nowak-Wegrzyn et al. [13] showed no significant differences in the serum wheat- and ω5-gliadin-specific IgE levels between the oral gluten immunotherapy group (52.2% tolerance) and placebo group (0% tolerance); however, serum ω5-gliadin-specific IgG_4_ levels increased significantly in the oral gluten immunotherapy group compared with those in the placebo group. We did not measure the serum ω5-gliadin-specific IgG_4_ level in this study. However, it can also play a role in inhibiting the binding of allergens to specific IgE.

The maximum amount of 1BS-18 Hokushin bread was set at 60 g to determine the safe consumption dose in this study. As 60 g of bread is equivalent to a typical serving of one slice of bread, the quality of life of patients with WDEIA may be maintained by serving 1BS-18 Hokushin wheat bread. Fourteen of the 16 patients with WDEIA completed step 1, resulting in a safe dose of 60 g. Two patients (SHIM010 and FUKU002) did not reach the safe dose of 60 g of 1BS-18 Hokushin wheat bread. The intolerance of SHIM010 to 60 g of 1BS-18 Hokushin wheat bread may be due to a high level of gluten-specific IgE, resulting in a reaction with other gluten proteins in 1BS-18 Hokushin bread. Intolerance to FUKU002 may be due to sensitization against nongluten proteins, such as lipid transfer proteins since the basophil activation test of the patient showed low-level reactions to all gluten fractions (Table 2).

Bread seems to be advantageous in long-term studies because of the high adherence of patients. In this study, 14 of the 16 patients completed 12 weeks of continuous bread ingestion (Table 3). Although 2 (SHIM010 and HIRO009) of the 16 patients had allergic reactions during the 12 weeks-continuous ingestion of the bread, their allergic scores were 2 and 3, respectively (Table 3), which recovered in a short time, supporting the low allergenicity of 1BS-18 Hokushin bread.

A limitation of this study is that only 16 patients were enrolled. However, data published to date on wheat oral immunotherapy in adult patients with WDEIA are scarce. Most oral immunotherapy studies on wheat allergy have been performed on relatively small numbers of young subjects. Sato et al. reported the outcomes of wheat oral immunotherapy in 18 subjects of age ranging from 5.9 to 13.6 years old. Nowak-Wegrzyn et al. enrolled 23 subjects of age ranging from 4.6 to 18.4. Sugiura et al. treated 32 subjects aged 4 to 6 years. Moreover, a large-scale study in adult patients is difficult compared with that in pediatric patients, since the prevalence of WDEIA in adults is lower than that of wheat allergy in children [4].

We have previously reported that treatment of adult patients with WDEIA with omalizumab reduced allergic reactions upon consuming wheat products [10]. However, 1 year of omalizumab treatment did not induce sustained unresponsiveness to wheat, and the inhibitory effects were lost after stopping omalizumab in both the basophil activation test and allergic reaction of the patients to wheat products. In the omalizumab study, 15 patients safely consumed wheat products in their daily lives for approximately 36 weeks. However, 13 of the 15 patients developed allergic reactions upon ingesting wheat products after stopping the omalizumab treatment. The failure of desensitization may be because the amount of ingested wheat products was insufficient, or it was consumed irregularly rather than daily by patients.

Therefore, the present study was conducted to examine the effect of daily consumption of 60 g of bread, equivalent to a typical serving of one slice of bread or the maximum safe amount for each subject, on desensitization in adult patients with WDEIA. A major strength of our study was the use of hypoallergenic 1BS-18 Hokushin bread for a homogeneous population of patients sensitized to ω5-gliadin so that the patients could safely ingest relatively large amounts of bread. It is speculated that 1BS-18 Hokushin bread contains fewer IgE epitopes but sufficient T cell epitopes, the key to inducing desensitization, based on our previous study using an experimental rat wheat allergy model [8, 9].

Another limitation of this study was the lack of a control bread. As WDEIA is a relatively severe form of food allergy often associated with anaphylactic shock, it is risky for patients to consume up to 60 g of bread at home. Thus, only the 1BS-18 Hokushin bread was used in the preliminary clinical study.

In conclusion, approximately 80% of patients with WDEIA safely consumed 1BS-18 Hokushin bread up to 60 g for 12 weeks; however, this consumption is not sufficient to obtain desensitization in these patients. (2886 words)

## Acknowledgements

This work was partially supported by the JSPS KAKENHI (Grant Numbers 19K17799, 19K08791, and 20K08802), LOTTE Foundation, and the Practical Research Project for Allergic Diseases and Immunology (Research on Allergic Diseases and Immunology) of the Japan Agency for Medical Research and Development. We thank Mrs. Kiyoe Ueda for her technical assistance with the basophil activation test. We thank Takahiro Okabe, MD, for his support with subject recruitment.

## Conflicts of interest

MH received lectures and consultations from Novartis. The other authors declare no conflicts of interest.

## Author contributions

KK, YC, and EM designed the study. YC, AS, RK, MO, MH, YO, AF, and EM contributed to data collection. KK, RS, and EM performed the statistical analyses and interpreted the results. All authors read, revised, and approved the final manuscript.

## Supplementary material

Supplementary Tables 1–4 can be found via 10.5415/apallergy.2022.12.e38

Supplementary Tables 1–4

Click here to view
